# The Third Delay in General Surgical Care in a Regional Referral Hospital in Soroti, Uganda

**DOI:** 10.1007/s00268-022-06591-0

**Published:** 2022-05-26

**Authors:** Savannah Starr, Woon Cho Kim, Rasheedat Oke, Melissa Carvalho, Yera Ledesma, Silas Okullu, Mary Goretty Ariokot, Andrew Hyginus Wange, Esther Agwang, Peter Ekuchu, Marissa Boeck, Catherine Juillard, Mary Margaret Ajiko, Rochelle A. Dicker

**Affiliations:** 1 David Geffen School of Medicine, University of California at Los Angeles, Los Angeles, CA USA; 2 Department of Surgery, University of California San Francisco, San Francisco, CA USA; 3 Program for the Advancement of Surgical Equity, Department of Surgery, University of California, 10833 Le Conte Avenue, 72160 CHS, Los Angeles, CA 90095 USA; 4 Department of Surgery, Soroti Regional Referral Hospital, Soroti, Uganda

## Abstract

**Background:**

Building capacity for surgical care in low-and-middle-income countries is essential for the improvement of global health and economic growth. This study assesses in-hospital delays of surgical services at Soroti Regional Referral Hospital (SRRH), a tertiary healthcare facility in Soroti, Uganda.

**Methods:**

A prospective general surgical database at SRRH was analyzed. Data on patient demographics, surgical characteristics, delays of care, and adverse clinical outcomes of patients seen between January 2017 and February 2020 were extracted and analyzed. Patient characteristics and surgical outcomes, for those who experienced delays in care, were compared to those who did not.

**Results:**

Of the 1160 general surgery patients, 263 (22.3%) experienced at least one delay of care. Deficits in infrastructure, particularly lacking operating theater space, were the greatest contributor to delays (*n* = 192, 73.0%), followed by shortage of equipment (*n* = 52, 19.8%) and personnel (*n* = 37, 14.1%). Male sex was associated with less delays of care (OR 0.63) while undergoing emergency surgeries (OR 1.65) and abdominal surgeries (OR 1.44) were associated with more frequent delays. Delays were associated with more adverse events (10.3% vs. 5.0%), including death (4.2% vs. 1.6%). Emergency surgery, unclean wounds, and comorbidities were independent risk factors of adverse events.

**Discussion:**

Patients at SRRH face significant delays in surgical care from deficits in infrastructure and lack of capacity for emergency surgery. Delays are associated with increased mortality and other adverse events. Investing in solutions to prevent delays is essential to improving surgical care at SRRH.

## Introduction

Low- and middle-income countries (LMICs) face an urgent need for accessible and safe surgical care. An estimated 30% of the global disease burden results from conditions that can be treated surgically [1]. Despite representing 71% of the total global population, less than a third of operations worldwide are performed in LMICs [2]. There is an economic impetus to improve surgical capacity globally- an estimated US $20.7 trillion of global economic losses from 2015 to 2030 are thought to be attributable to surgical conditions, with half coming from LMICs [3]. Scaling up surgical care in LMICs alone could prevent 116.1 million disability-adjusted life years (DALYs) annually, surpassing the unaddressed global burdens of HIV/AIDS, tuberculosis, or malaria [4]. Therefore, ensuring an accessible and functional surgical system in LMICs is a priority in improving health and wealth locally and globally.

Uganda is a country in Eastern sub-Saharan Africa in which almost a third of its population lives in poverty [5]. Uganda is estimated to have approximately one surgeon per 100,000 people and only 0.2 major operating theaters per 100,000 people [5], which is in stark contrast to North America with 14.3 operating theaters per 100,000 people [6]. A nationwide cross-sectional study found that while 20.2% of Ugandans will experience a condition requiring surgery in their lifetime, 10.2% of the population will be unable to access it [7]. This represents an estimated 3.6 million people with unmet surgical needs that can be treated by improving surgical access and capacity [7].

Improving surgical systems requires a thoughtful examination of the underlying causes of inaccessibility that patients may experience in the pre-and in-hospital setting. A 2015 Lancet report on improving surgical care globally presented the “Three Delays” framework to characterize types of delays between symptom onset and appropriate care in order to pinpoint opportunities in patient trajectory to optimally target interventions that reduce delays in care [8]. The “Third Delay” encompasses barriers to receiving care once the patient has accessed the hospital, such as due to inadequate hospital resources or infrastructure, such as shortage of electricity, running water, and oxygen as well lack of essential medications, imaging, pathology, and a safe blood supply [8]. In this study, we examine causes of the “Third Delay”, or in-hospital delays of care, for general surgical patients at Soroti Regional Referral Hospital (SRRH), a tertiary level healthcare facility in Soroti, Uganda. We hypothesized that several patients with surgical conditions face delays in receiving care due to inadequate hospital resources or infrastructure, and these delays lead to increased morbidity and mortality.

## Materials and methods

### Study setting

This study was conducted at Soroti Regional Referral Hospital (SRRH), one of thirteen regional hospitals in Uganda. It is a government-run 250-bed hospital that serves 21,000 inpatients and 103,000 outpatients yearly. It has one operating theater with two operating tables, where two operations can be occurring simultaneously [9]. A median of 32.5 operations is done monthly. SRRH serves a catchment area of approximately two million people (5% of the Ugandan population) [9]. There are two attending general surgeons, two attending gynecological surgeons, and three nurse anesthetists [9]. Representing the second-highest level of care within the national health system, SRRH is the main referral center for specialized surgical care in the Teso sub-region [10].

### Data collection and organization

A general surgical registry was established at SRRH in 2017 (See Appendix 1). A registered nurse was trained and served as registrar who prospectively obtained data from patients, managing team, and medical records for the study. Data were collected on paper forms for patients undergoing operations for general surgical conditions from January 2017 to February 2020. The data were then entered into REDCap [11], a secure electronic database hosted at the University of California San Francisco and University of California Los Angeles, by the registrar. Data verification for completeness and accuracy was conducted before and after data entry into REDCap. Patients with obstetrical and traumatic diseases were excluded, as these data were collected separately.

Delays were defined by the providers caring for each patient, who evaluated times of arrival, time of decision to operate, and time of operation to determine if the patient had an in-hospital surgical delay based on the procedure taking place and clinical condition of the patient. Providers then reported which factors caused the delay and this information was recorded in our registry. The various causes of care delays were subsequently organized into broad categories to represent deficits in personnel, equipment, and infrastructure.

### Statistical analysis

Patients were divided into two study populations: patients with reported in-hospital delays and patients without reported delays. Patient demographics and operation characteristics were compared. Adverse outcomes, a composite variable defined by the patient incurring a complication, death, and/or new long-term disability, were also extracted and compared.

Descriptive analyses were presented as medians and interquartile range (IQR) for continuous variables, and categorical variables were presented as frequencies and proportions. Univariate analysis between cohorts was performed using Chi-square analysis or Fisher’s exact tests for categorical variables. Continuous variables were analyzed using *t*-test and Mann–Whitney *U* test for parametric and nonparametric variables, respectively.

Multivariate logistic regression models were created to identify significant factors associated with care delays and adverse events for general surgery patients at SRRH. Variables included in the delays model were those with statistically significance on bivariate analysis along with age and sex. Variables included in the adverse events model included delays, age, sex, and variables captured in our registry that have been shown to affect rates of adverse events in prior studies, including emergency surgery [12], pre-operative comorbidities [13, 14], wound class [15], and antibiotic administration within 60 min of incision [16].

Patients with missing data were excluded from analysis. 4.1% of patients had missing data for the variables analyzed. All statistical tests were 2-sided and differences were considered significant when *p* ≤ 0.05. All statistical analyses were performed with SPSS statistical software [17].

## Results

### Demographic comparisons for patients with or without delay

A total of 1160 general surgery patients were captured in the registry between January 2017 to February 2020 with 56% being male. Among these patients, 263 (22.7%) had a delay in receiving general surgical care after hospital arrival although some incurred multiple delays (*n* = 174). There were no significant differences in median age between patients who experienced delays of care and those who did not (27 [IQR: 10–48.5] vs. 29 [IQR: 8–56], *p* = 0.77). Male patients were less likely to have a delay of care (50.2% vs. 57.9%, *p* = 0.02). (Table 1).

**Table 1:** Patient, surgical, and hospital course characteristics of general surgery patients at SRRH (*n* = 1160^a^)

Characteristics	All patients (*n* = 1160)	No delay (*n* = 897)	Delay (*n* = 263)	*p*-value
Age (median, IQR) (*n* = 1157)	28 (8–49)	29 (8–56)	27 (10–48.5)	0.77
Male (*n*, %) (*n* = 1160)	650 (56.0%)	519 (57.9%)	132 (50.2%)	0.02*
Comorbidities (*n*, %) (*n* = 1160)	99 (8.5%)	71 (7.9%)	28 (10.6%)	0.17
Referred from elsewhere (*n*, %) (*n* = 1154)	109 (9.4%)	81 (9.1%)	28 (10.7%)	0.47
*Anatomic region operated on *(*n*, %) (*n* = 1160)				0.08
Head and neck	36 (3.1%)	26 (3.0%)	10 (3.8%)	0.43
Skin and subcutaneous tissue	267 (23.0%)	209 (23.3%)	58 (22.1%)	0.74
Abdomen	480 (41.4%)	355 (39.6%)	125 (47.5%)	< 0.01*
Genitourinary	264 (22.8%)	218 (24.3%)	46 (17.5%)	0.01*
Musculoskeletal	80 (7.0%)	66 (7.4%)	14 (5.3%)	0.33
Thorax	4 (0.3%)	4 (0.4%)	0 (0.0%)	0.58
Vascular	3 (0.3%)	3 (0.3%)	0 (0.0%)	1.00
Hospital length of stay (days, mean, IQR) (*n* = 1155)	8.0 (2.00–9.00)	7.63 (2.00–9.00)	9.13 (2.00–11.00)	0.03*
Emergency surgery (*n*, %) (*n* = 1157)	225 (19.4%)	153 (17.1%)	72 (27.4%)	< 0.01*

^*^Indicates statistical significance (*p* < 0.05)

^a^ Variables had missing data; hence, the total *n* differs for each variable. Missing data were excluded from the analysis

### Surgical characteristic and hospital course comparisons for patients with or without delay

Abdominal surgeries were more associated with delays (47.5% vs. 39.6%, *p* < 0.01) while genitourinary surgeries were associated with less (17.5% vs. 24.3%, *p* = 0.01). Median hospital length of stay for patients without a delay of care was 7.63 days compared to 9.13 days for those with a delay (*p* = 0.03). Surgeries classified as emergencies were significantly associated with more delays of care versus scheduled surgeries (27.4% vs. 17.1%, *p* < 0.01) (Table 1).

### Reasons for in-hospital delays in surgical care

**Fig. 1:**
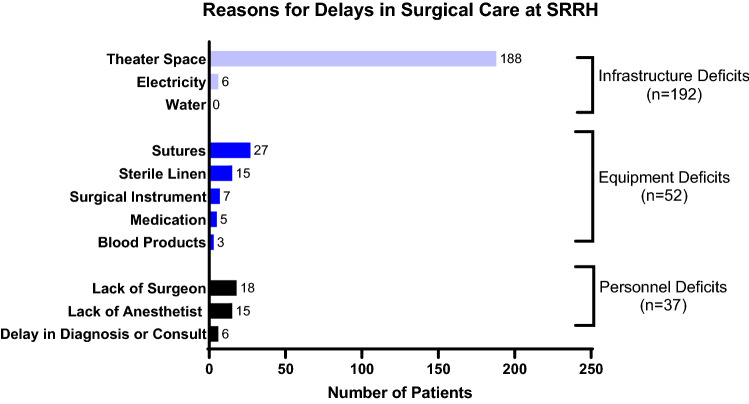
Reasons for in-hospital delays in care at Soroti Regional Referral Hospital (SRRH) (*n* = 263). *some patients incurred multiple delays in care (*n* = 174)

Among patients receiving delayed care, 192 (73.0%) experienced delays due to deficits in infrastructure. Lack of theater space was the largest contributor to infrastructure and total delays, making up 71.4% of total delays of care. Patients also experienced delays from deficient electricity (2.3%) but not water (0%) (Fig. 1).

Following infrastructure, equipment deficits were the next largest contributor to delays (19.8%). The equipment most needed was sutures, which was seen in 10.3% of patients experiencing delay. Lack of sterile linen (5.7%), surgical instruments (2.7%), medications (1.9%), and blood products (1.1%) also conferred delays in surgery (Fig. 1).

Last, personnel deficits were seen in 14.1% of patients who had a delay. The prominent staffing delay was due to a lack of surgeon (6.8%), but lack of anesthetist (5.7%) was also seen. Patients also experienced impediments in timely care due to a delay in consult and diagnosis (2.3%) (Fig. 1).

### Factors associated with delays in care

**Fig. 2:**
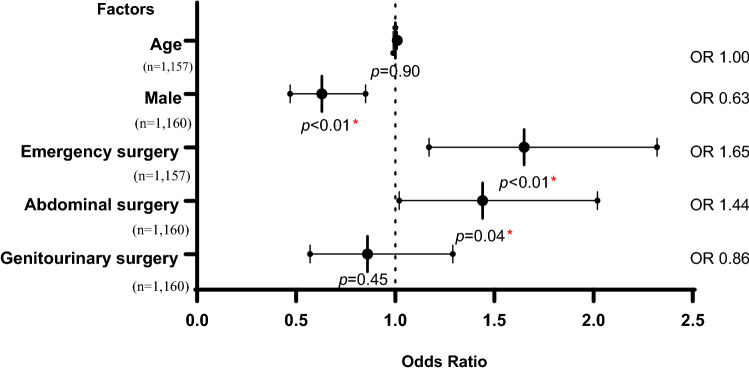
Multivariate logistic regression for factors associated with delays of care among general surgery patients at SRRH (*n* = 1154^a^). ^a^Variables had missing data; hence, the total n differs for each variable. Missing data were excluded from the analysis. *Indicates statistical significance (*p* < 0.05)

In multivariate logistic regression, compared to female patients, male patients had 37% lower odds of having a delay in surgical care (OR 0.63). Conversely, patients undergoing emergency surgery (OR 1.65) and abdominal surgery (OR 1.44) had increased odds of having a delay, compared to patients undergoing elective and non-abdominal surgery, respectively (Fig. 2).

### Emergent versus elective surgeries

Nearly one-third (32.0%) of patients undergoing emergency surgery experienced a delay in care compared to 20.4% of patients undergoing elective surgery (*p* < 0.01). There was no significant difference between elective and emergent surgeries with regard to delays due to deficits in infrastructure (*p* = 0.13); however, emergency surgery was associated with delays due to equipment and personnel (*p* < 0.01 and *p* < 0.01, respectively) (Table 2).

**Table 2:** Delays of care seen for elective versus emergent surgeries (*n* = 1,157)

Type of delay	Elective (*n* = 932)	Emergent (*n* = 225)	*p*-value
Any delay (*n* = 262)	190 (20.4%)	72 (32.0%)	< 0.01*
Infrastructure (*n* = 192)	147 (15.8%)	45 (20.0%)	0.13
Equipment (*n* = 52)	32 (3.4%)	20 (8.8%)	< 0.01*
Personnel (*n* = 37)	15 (1.6%)	22 (9.8%)	< 0.01*

Variables had missing data; hence, the total *n* differs for each variable. Missing data were excluded from the analysis

^*^Indicates statistical significance (*p* < 0.05)

For emergency cases, the time required to get a patient to the operating theater was compared between cohorts. Patients with delays had lower rates of arriving at the operating theater within an hour (15.0% vs. 36.7%, *p* < 0.01) and higher rates of arriving within 6–12 h (23.3% vs. 10.0%, *p* = 0.02) and greater than 24 h (6.7% vs. 0.8%, *p* = 0.03) of arrival (Table 3).

**Table 3:** Time to the operating theater for emergency general surgery cases at SRRH (*n* = 180)

Time to theater for emergency surgery	All patients (*n* = 180)	No delay (*n* = 120)	Delay (*n* = 60)	*p*-value
<1 h	53 (29.4%)	44 (36.7%)	9 (15.0%)	<0.01*
1–6 h	88 (48.9%)	59 (49.2%)	29 (48.3%)	0.92
6–12 h	26 (14.4%)	12 (10.0%)	14 (23.3%)	0.02*
12–24 h	8 (4.4%)	4 (3.3%)	4 (6.6%)	0.31
>24 h	5 (2.8%)	1 (0.8%)	4 (6.7%)	0.03*

Variables had missing data; hence, the total *n* differs for each variable. Missing data were excluded from the analysis

^*^Indicates statistical significance (*p* < 0.05)

### Adverse outcomes associated with delays in care

Patients who had any delay in surgical care were associated with increased rates of adverse outcomes (*p* =  < 0.01). In the breakdown of each adverse outcome, delays in care were significantly more associated with complications (*p* = 0.01) and death (*p* = 0.02). Increased rates of a newly-acquired long-term disability were not seen (*p* = 0.32) (Table 4).

**Table 4:** Adverse outcomes based on status of in-hospital delays of care (*n* = 1,160)

Outcome	All patients (*n* = 1160)	No delay (*n* = 897)	Delay (*n* = 263)	*p*-value
Any adverse outcome	72 (6.2%)	45 (5.0%)	27 (10.3%)	< 0.01*
Complications	52 (4.5%)	32 (3.6%)	20 (7.6%)	0.01*
Death	25 (2.2%)	14 (1.6%)	11 (4.2%)	0.02*
Newly acquired long-term disability	23 (2.0%)	16 (1.8%)	7 (2.7%)	0.32

^*^Indicates statistical significance (*p* < 0.05)

**Fig. 3:**
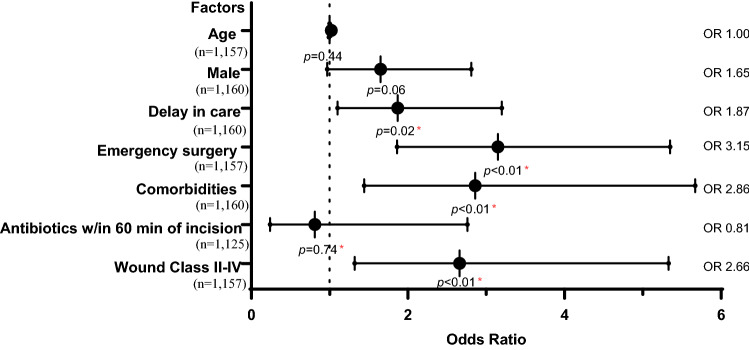
Multivariate logistic regression analysis of adverse outcomes among general surgery patients at SRRH (*n* = 1119^a^). ^a^Variables had missing data; hence, the total *n* differs for each variable. Missing data were excluded from the analysis. *Indicates statistical significance (*p* < 0.05)

Results of multivariate logistic regression revealed that delays in surgical care (OR 1.87), emergency surgery (OR 3.15), comorbidities (OR 2.86), and Class II–IV incision site wounds (OR 2.66) were all significantly associated with postsurgical adverse events (Fig. 3).

## Discussion

General surgical patients at Soroti Regional Referral Hospital (SRRH) face impediments in timely surgical care and patients who do experience an in-hospital care delay have more complications and higher mortality, emphasizing the need to address causes of these delays. Patients at SRRH are facing delays from multiple facets of the healthcare system, including limitations of hospital infrastructure, equipment, and personnel. A previous mixed methods study at SRRH interviewed healthcare workers regarding their perceived barriers to quality surgical care, which identified lack of space, patient overload, inadequate equipment, and inadequate processes as major challenges [18]. The deficits described by these healthcare workers were also illustrated and quantified in this study. Furthermore, we demonstrated these challenges to care to have consequences on patient outcomes.

Lacking theater space was revealed to be the largest contributor to delays in surgical care in our study. SRRH has a catchment area encompassing two million people but contains only two operating tables in one operating theater [9]. This equates to only 0.1 operating tables and 0.05 operating theaters per 100,000 Ugandans in the Soroti region, far below the average of 2 operating rooms per 100,000 people in LMICs and 14 operating rooms per 100,000 people in high-income countries (HICs) [19]. This study demonstrated that this deficit in surgical space results in delays in care at SRRH, which portends adverse outcomes. Investing in OR space in Uganda has also previously been shown to be cost-effective: the cost of implementing a dedicated pediatric OR at Naguru Hospital in Kampala, Uganda was shown to lie under both the WHO and the World Bank cost-effectiveness thresholds at $2321 per life saved [20]. Financial investment in development of infrastructure is essential to addressing the burden and outcomes of global surgical disease.

Our study also demonstrated that emergency general surgery at SRRH was associated with more in-hospital delays and adverse events, suggesting a need to improve the process of emergency surgical care delivery. The WHO’s 68th World Health Assembly established a resolution urging member states to establish and strengthen systems in the area of emergency surgery, recognizing that improvement of emergency surgical care has been neglected, but is highly cost-effective [21]. Additionally, a nationwide survey of Uganda’s public hospitals demonstrated that 73% of surgeries were performed on an emergency basis, further underscoring the need to improve timely emergency surgery to improve post-surgical outcomes [22]. Personnel deficits were the biggest contributor to delays in emergency surgery in this study, likely due to inability to staff operating rooms on short notice. Training and hiring more staff and implementation a robust call schedule could potentially mitigate delays and improve quality of emergency care at SRRH.

Comparing patient demographics among those facing delays, men faced fewer delays in surgical care than women. This highlights a sex disparity in timely surgical care, which has been demonstrated in other LMIC settings [23, 24]. Women in the general surgical population at SRRH facing more delays in surgical care could put them at higher risk for adverse events, though this was not directly assessed in this study. Future studies to determine prevalence of adverse events and the driving factors of sex disparities in hospital care should be conducted to provide more insight.

Several patient factors were not measured in this study which could provide more context about the characteristics of patients experiencing in-hospital delays. This includes socioeconomic indicators such as income, occupation, household size, readiness of cash, etc., or situational factors such as patient preference or hesitation. These factors could be addressed in further iterations of data collection at SRRH to strengthen our understanding of in-hospital delays in surgical care.

### Limitations

This study had several limitations. This was a single-center study and, therefore, the ability to generalize its results is limited. Delays were defined by surgeons/providers and could be susceptible to bias and underreporting of personnel delays. Times of arrival and surgery were also not captured in our tool, which limits our assessment of the severity of delays. Our conclusions are also limited due to our database not including variables commenting on the general condition of patients before and after surgery or barriers to seeking or reaching care. This could confound our conclusion that complications and death are due to in-hospital delays in care rather than the state of the patient prior to arriving at the hospital.

## Conclusions

Building capacity for surgical care in LMICs is essential for the improvement of global health and economic growth. Patients at Soroti Regional Referral Hospital in Soroti, Uganda face significant delays in receiving surgical care, particularly due to critical deficits in hospital infrastructure. Delivering quality emergency surgical care is essential in Uganda where conditions are more likely to progress to an emergency before care is accessed. At SRRH, patients undergoing emergency surgeries face more delays in care compared to patients undergoing elective surgery. Moreover, delays in care were associated with increased mortality and complications. Addressing root causes of delays in care is essential in improving surgical care for potentially two million people. This study unveils the deficits affecting surgical care at SRRH and demonstrates the need to allocate resources towards preventing these delays by investing in theater space, equipment, and training of medical professionals.
